# The Macrophage Scavenger Receptor A Is Host-Protective in Experimental Meningococcal Septicaemia

**DOI:** 10.1371/journal.ppat.1000297

**Published:** 2009-02-13

**Authors:** Annette Plüddemann, J. Claire Hoe, Katherine Makepeace, E. Richard Moxon, Siamon Gordon

**Affiliations:** 1 Sir William Dunn School of Pathology, University of Oxford, Oxford, United Kingdom; 2 Department of Paediatrics, Weatherall Institute of Molecular Medicine, John Radcliffe Hospital, Oxford, United Kingdom; Northwestern University Feinberg School of Medicine, United States of America

## Abstract

Macrophage Scavenger Receptor A (SR-A) is a major non-opsonic receptor for *Neisseria meningitidis* on mononuclear phagocytes *in vitro*, and the surface proteins NMB0278, NMB0667, and NMB1220 have been identified as ligands for SR-A. In this study we ascertain the *in vivo* role of SR-A in the recognition of *N. meningitidis* MC58 (serogroup B) in a murine model of meningococcal septicaemia. We infected wild-type and SR-A^−/−^ animals intraperitoneally with *N. meningitidis* MC58 and monitored their health over a period of 50 hours. We also determined the levels of bacteraemia in the blood and spleen, and measured levels of the pro-inflammatory cytokine interleukin-6 (IL-6). The health of SR-A^−/−^ animals deteriorated more rapidly, and they showed a 33% reduction in survival compared to wild-type animals. SR-A^−/−^ animals consistently exhibited higher levels of bacteraemia and increased levels of IL-6, compared to wild-type animals. Subsequently, we constructed a bacterial mutant (MC58-278-1220) lacking two of the SR-A ligands, NMB0278 and NMB1220. Mutation of NMB0667 proved to be lethal. When mice were infected with the mutant bacteria MC58-278-1220, no significant differences could be observed in the health, survival, bacteraemia, and cytokine production between wild-type and SR-A^−/−^ animals. Overall, mutant bacteria appeared to cause less severe symptoms of septicaemia, and a competitive index assay showed that higher levels of wild-type bacteria were recovered when animals were infected with a 1∶1 ratio of wild-type MC58 and mutant MC58-278-1220 bacteria. These data represent the first report of the protective role of SR-A, a macrophage-restricted, non-opsonic receptor, in meningococcal septicaemia *in vivo*, and the importance of the recognition of bacterial protein ligands, rather than lipopolysaccharide.

## Author Summary

Macrophages are innate immune cells that provide a first defence against infection. Several receptors on the surface of macrophages mediate recognition of invading pathogens, and one of these is the Macrophage Scavenger Receptor A (SR-A). SR-A recognises *Neisseria meningitidis*, a bacterium that causes meningitis and septic shock, via proteins on the surface of the bacterium. In this study we investigated the interaction of SR-A with *N. meningitidis* in a mouse model for septic shock, by infecting mice with *N. meningitidis* and comparing a mouse strain expressing SR-A with one that does not. The health of mice not expressing SR-A deteriorated more rapidly and fewer animals survived compared to those expressing SR-A. Mice lacking SR-A had higher numbers of bacteria in their blood and also produced more cytokines that can cause septic shock. We also infected mice with bacteria that did not express two of the proteins recognised by SR-A. In this case, no differences in survival, levels of bacteria, or cytokines were detected between animals that expressed SR-A and those that did not. Therefore, we show that the macrophage receptor SR-A is protective in the development of septic shock induced by *N. meningitidis*.

## Introduction

The innate immune system is a first line of defence against invading pathogens and macrophages play an integral role in the innate immune defence against bacterial infection. This is based on the recognition of conserved microbial structures, termed pathogen-associated molecular patterns (PAMPs), by a range of pattern recognition receptors (PRRs). Macrophages (Mϕ) express different classes of innate PRRs with diverse functions, including the phagocytic Scavenger receptors (SRs) [1]. One of the receptors belonging to this family is the class A scavenger receptor (SR-A), which has been shown to recognise a range of polyanionic molecules [2]. SR-A is a trimeric type II transmembrane glycoprotein consisting of a cytoplasmic tail, transmembrane domain, spacer region, α-helical coiled coil domain, collagenous domain and C-terminal cysteine-rich domain. SR-A expression is mostly restricted to macrophages and is not found on polymorphonuclear neutrophils or monocytes [3]. Recently, the expression of SR-A was shown on mast cells [4] and specific sub-populations of bone marrow-derived dendritic cells (DC) and splenic DC [5]. Selected endothelial cells and smooth muscle cells within atherosclerotic plaques also express SR-A [6].

The Scavenger receptors play an important role in microbial recognition and clearance, and SR-A has been shown to bind both Gram-positive and Gram-negative bacteria [7]. Hampton and colleagues first proposed that SR-A might be involved in antimicrobial host defence, based on their observation that SR-A could bind lipid A, an integral part of lipopolysaccharide (LPS) [8]. Subsequent studies showed that SR-A also recognises the Gram-positive cell-wall component lipoteichoic acid (LTA) [9]. Furthermore, SR-A binds to different LTA structures with varying specificity depending on their exposed negative surface charge. Unmethylated bacterial CpG DNA, another major immunostimulatory microbial product, is also recognised by SR-A [10]. Using a range of Gram-positive organisms, Dunne and co-workers confirmed that both soluble and cell-associated forms of SR-A are not only able to bind bacterial components, but can also recognise intact live organisms [11]. SR-A has been shown to play a role in both infection and inflammation. *In vivo* studies with three Gram-positive organisms have shown that SR-A^−/−^ mice are more susceptible to infection. SR-A^−/−^ animals exhibited deficient clearance of bacteria from the liver and spleen in experimental *Listeria monocytogenes* infection [12]. SR-A^−/−^ mice also showed increased susceptibility to *Staphylococcus aureus* and *Streptococcus pneumoniae* infection [13],[14]. A possible anti-inflammatory host-protective role of SR-A was proposed by Haworth et al., who observed that SR-A^−/−^ mice formed normal granulomas in response to BCG (Bacille Calmette-Guérin) priming [15]. However, these animals were more susceptible to endotoxic shock as a result of increased pro-inflammatory cytokine secretion in response to additional lipopolysaccharide (LPS) challenge. In addition, SR-A has been shown to modulate chemokine levels in specific acute inflammatory conditions to ensure an inflammatory response of the appropriate magnitude [16].

*Neisseria meningitidis* is a Gram-negative obligate commensal bacterium that colonises the human nasopharynx, however when the bacterium crosses this barrier, it causes meningitis and rapid septicaemia, particularly in young children and teenagers. We have shown previously that uptake of *N. meningitidis* by macrophages is mediated almost exclusively via SR-A [17]. Interestingly, experiments employing an *N. meningitidis lpxA* mutant revealed that recognition of *N. meningitidis* by SR-A was independent of lipopolysaccharide, and we identified three bacterial surface protein ligands for SR-A, namely NMB0278, NMB0667 and NMB1220 [18]. In this study we investigated the *in vivo* role of SR-A in inflammation in a murine meningococcal septicaemia model. We also ascertained the contribution of the identified surface protein ligands by constructing bacterial mutants in the SR-A ligands and examining the effects of a double mutant *in vivo*. We show that SR-A knock-out mice are more susceptible to septicaemia induced by *N. meningitidis* than wild-type mice, and that for double knock-out bacteria lacking two SR-A ligands, these effects are abrogated.

## Materials and Methods

### Chemicals and Reagents

Unless otherwise stated, all chemicals were from Sigma (Poole, United Kingdom). Acetylated low density lipoprotein (AcLDL) and Rhodamine Green X (RdGnX) were obtained from Molecular Probes (Eugene, OR, USA). The TMB substrate reagent set was purchased from BD Biosciences Pharmingen (San Diego, CA). All culture media were from Gibco (Paisley, United Kingdom). The rat monoclonal anti-CD68 monoclonal antibody FA-11 was obtained from AbD Serotec (Kidlington, UK). The rat monoclonal antibody against the 7/4 murine differentiation antigen was generated in this laboratory [19]. M5114, the rat monoclonal antibody recognising murine MHC-II was obtained from R&D Systems (Abingdon, UK).

### Bacterial Strains and Culture Conditions

The *Neisseria meningitidis* strains used in this study are listed in Table 1. All strains were grown overnight at 37°C on brain-heart infusion (BHI) medium (Oxoid), supplemented with Levinthal's reagent (10% vol/vol) and solidified with agar (1% [wt/vol]; Bioconnections), in an atmosphere of 5% CO_2_. For selection of strains following transformation, kanamycin (75 µg ml^−1^) or erythromycin (6 µg ml^−1^) was added to the culture medium. *Escherichia coli* strain DH5α was used to propagate recombinant DNA constructs and was grown at 37°C on Luria-Bertani (LB) medium supplemented with kanamycin (50 µg ml^−1^), erythromycin (300 µg ml^−1^) or ampicillin (50 µg ml^−1^) where appropriate. For fluorescent labelling, *N. meningitidis* were fixed with 70% ethanol and labelled with RdGnX according to the manufacturer's instructions.

**Table 1:** List of *N. meningitidis* strains and plasmids used in this study.

*N. meningitidis* and Plasmids	Description	Source or Reference
MC58	L3 immunotyping reference strain, Serogroup B genome sequenced strain	[37]
MC58-278	NMB0278 gene disrupted	This study
MC58-1220	NMB1220 gene disrupted	This study
MC58-278-1220	NMB0278 and NMB1220 genes disrupted	This study
pT7Blue	Cloning vector	Novagen
pER2	Source of erythromycin resistance cassette	[26]
pUC4-kan	Source of kanamycin resistance cassette	
pT7-278-kan	Disrupted NMB0278 gene from MC58, cloned into pT7Blue	This study
pT7-667-ery	Disrupted NMB0667 gene from MC58, cloned into pT7Blue	This study
pT7-1220-ery	Disrupted NMB1220 gene from MC58, cloned into pT7Blue	This study

### DNA Techniques

Recombinant DNA techniques were performed as described by Sambrook *et al.* [20]. Restriction endonuclease and DNA modifying enzymes were obtained from Boehringer Mannheim or New England Biolabs and used according to the manufacturers' instructions. Oligonucleotide primers were synthesised by Sigma-Genosys. Standard polymerase chain reaction (PCR) amplifications were performed in 50 µl reaction volumes (final concentrations: 20 mM Tris-HCl, pH 8.4; 50 mM KCl; 2.5 mM MgCl_2_; 0.4 µM forward primer; 0.4 µM reverse primer; 0.4 mM dNTPs) with 1.25 U *Taq* recombinant polymerase (Invitrogen, Paisley, UK) in a Master-Cycler (Eppendorf) gradient thermal cycler. Thirty cycles of PCR were performed, each consisting of 1 min denaturation at 94°C, 1 min annealing at typically 5°C below Tm and 1 min extension at 72°C, with a final prolonged extension of 10 min at 72°C. Chromosomal DNA was prepared from *N. meningitidis* strains as described previously [21]. A list of oligonucleotide primers used in this study is given in Table 2.

**Table 2:** List of oligonucleotide primers used in this study.

Primer name	Sequence (5′→ 3′)	Gene in which primer binds
278-f	AGA CAC CTT GCC CTC GGC	NMB0278
278-r	TTC GCG TAC TTT GTC CGC C	NMB0278
278-out-r	TAT CGC GCC GTT ATG CCG	NMB0279
278-out-f	AAA GAT TGT GCG AAC AGC C	NMB0278
Kan-5-out	TCA AAA ATA TGG TAT TGA TAA T	*KanR* [pUC4-kan]
Kan-3-out	TGT AAC ATC ATT GGC AAC GC	*KanR* [pUC4-kan]
667-f	ATC GTA CTG TTT CTA GCT GTC G	NMB0667
667-r	ATG CGG TTT TGC CGC CCG	NMB0667
667-f2	TCG CCG TTG TCG CCT AC	NMB0667
667-out-f	ACA CTG CTG GTC AGA CCC TGC	NMB0666
667-out-r	TGA ATG CG GAGA GTT CTT CC	NMB0667
ery-if	ACA TAA TAT AGA TAA AAT AAT GAC	*ermC* [pER2]
ery-ir	ATA ATT TAT AGC TAT TGA AAA GAG	*ermC* [pER2]
1220-f	TCA AAT CCT TTG TTG TCA TCC C	NMB1220
1220-r	TTT CAT ACC GGC AGA AAT CAG	NMB1220
1220-5f	ATA CGG GGC AAG AAG AGC TTG	NMB1220
1220-5r	TCG GTT ACT TGG AGA TCT ATG ATG CCG TC	NMB1220
1220-3f	TT CGA GCA ACT AGA TCT TGG CGA TTA CCC	NMB1220
1220-3r	TGA AGC GGA ATA CA ACCT TGC	NMB1220
ery-eag-f	GAT CCC CGG CCG TGC AGG AAT TCG ATA TCA AGC	*ermC* (pER2)
ery-eag-r	CCG GGC CGG CCG TCG TCG AGG TCG ACG GTA TCG	*ermC* (pER2)

### Tricine-SDS-PAGE

Outer membrane vesicles were prepared from *N. meningitidis* cells as described by Heckels and Williams et al [22],[23]. Briefly, cells were harvested from confluent growth of bacteria on 40 BHI plates into 0.2 M lithium acetate (40 ml, pH 5.8) and extracted at 45°C in the presence of 2 mm glass beads (20 ml). Live bacteria were removed by centrifugation at 13,00 g, 20 min, the supernatant was then subjected to a repeat of this step. The outer membranes were recovered by centrifugation at 11,000 g, 4°C, 2 h and the resuspended in 200 µl dH_2_O. The protein concentration of the outer membranes was determined by performing a Lowry MicroAssay (Sigma) according to the manufacturer's instructions. 15 µg of total protein was diluted in dH_2_O and loaded in each well. Whole cell lysates were prepared from *N. meningitidis* strains grown overnight, by harvesting and resuspending cells in PBS and then adding the equivalent amount of dissociation buffer (125 mM Tris, pH 6.8, 20% [v/v] glycerol, 3.9% [w/v] SDS, 10% β-mercaptoethanol, 0.04% [w/v] bromphenol blue). All samples were boiled at 100°C for 5 min. Samples were separated by tricine-sodium dodecyl sulphate-polyacrylamide gel electrophoresis (T-SDS-PAGE) using 16.5% gels run at 30 mA at 4°C for 18 hr [24], and were visualised by staining with silver nitrate according to the manufacturer's instructions (Amersham Biosciences).

### Disruption of Genes and Construction of Mutant Strains

To mutate the *N. meningitidis* NMB0278 gene, the gene was first amplified by PCR from strain H44/76 chromosomal DNA with oligonucleotide primers 278-f/278-r and cloned into the plasmid pT7Blue (Novagen). A kanamycin resistance (*kan^R^*) cassette was excised from pUC4-kan by digestion with *Hinc*II and inserted into the *Hinc*II site within the cloned NMB0278 gene. The resulting construct, pT7-278-kan was used to transform *N. meningitidis* strain MC58 as described previously [25]. Screening of transformants was performed by PCR using primers designed to bind within the *kanR* cassette and in the neighbouring gene to identify transformants containing a single, disrupted copy of NMB0278.

To mutate the *N. meningitidis* NMB0667 gene, the gene was first amplified by PCR from strain H44/76 chromosomal DNA with oligonucleotide primers 667-f/667-r and cloned into the plasmid pT7Blue (Novagen). An erythromycin resistance cassette (*ermC*) was excised from pER2 [26], by digestion with *Hinc*II and inserted into the *Sma*I site within the cloned NMB0667 gene. The resulting construct, pT7-667-ery, was used to transform *N. meningitidis* strain MC58. Transformants were screened by PCR using primers designed to bind within the *ermC* cassette and in the neighbouring gene to identify a transformant containing a single, disrupted copy of NMB0667.

To mutate the gene NMB1220, the 5′ and 3′ regions of the gene were first amplified by PCR from *N. meningitidis* strain MC58 chromosomal DNA, with oligonucleotide primers 1220-3f/1220-3r and 1220-5f/1220-5r, respectively, before cloning each product separately into pT7Blue, resulting in the plasmids pT7-1220-3 and pT7-1220-5. The *ermC* cassette was amplified from pER2 [26] with oligonucleotide primers ery-eag-f and ery-eag-r, digested with *Eag*I and inserted into the *Eag*I site present within the cloned 3′ region of the NMB1220 gene in pT7-1220-3 to give the construct pT7-1220-3-ery. The cloned 3′ region of NMB1220, interrupted with *ermC*, was excised from pT7-1220-3-ery with *Xba*I and *Bgl*II and inserted into the corresponding site within pT7-1220-5. The resulting construct, pT7-1220-ery, was used to transform *N. meningitidis* strain MC58. Transformants were screened by PCR using oligonucleotide primers 1220-f/1220-r designed to bind within NMB1220 to identify a transformant containing a single, disrupted copy of NMB1220.

### Resistance to the Bactericidal Effects of Human Sera

Bacteria cultured on BHI plates were assayed in pooled human serum as described previously [27].

### Cell Culture

Bone marrow-derived macrophages (BMMϕ) were prepared as described previously [7]. Mϕ were routinely cultured in RPMI supplemented with 100 U/ml penicillin, 100 µg/ml streptomycin and 2 mM L-glutamine (PSG), 10% foetal calf serum (FCS) and 15% L-cell conditioned medium.

### Uptake Assays

Bone marrow-derived macrophages were plated in 6-well bacteriological plastic dishes at a density of 1×10^6^ Mϕ per well 24 hours before use. Mϕ were washed twice in PBS and then incubated in Opti-MEM medium (Invitrogen, Paisley, UK) containing fluorescently-labelled bacteria as specified. After incubation with bacteria, the culture medium was removed and the cells washed three times with PBS. Cells for flow cytometry were harvested with PBS containing 10 mM EDTA and 4 mg/ml lidocaine-HCl and fixed with 4% (v/v) formaldehyde in PBS. Fluorescence was analysed on a FACScan (Becton Dickinson, Mountain View, CA) using the FL-1 or FL-2 photomultiplier where appropriate and the results analysed with CellQuest software. Results are representative of at least 3 independent experiments.

### Mouse Infection Studies

A murine intraperitoneal challenge model for bacterial clearance was employed [28]. C57BL/6J wild-type mice and a corresponding SR-A^−/−^ knock-out mouse strain were used. SR-A^−/−^ animals were developed and bred onto C57BL/6J background using standard molecular biology techniques [12]. All animals were bred and housed under specific pathogen-free conditions.

Meningococcal strains were grown overnight at 37°C in 5% CO_2_ on BHI plates as described above. Muller Hinton broth (8 ml) supplemented with 0.25% (w/v) glucose in a 50 ml tissue culture flask was inoculated with 1.2×10^9^ cfu from the overnight growth resulting in an initial OD_620_ of ∼0.1. The flask was incubated horizontally on a gently rocking platform at 37°C in 5% CO_2_ and bacteria were cultured to mid-logarithmic growth phase, defined as OD_620_ of ∼0.5 (approximately 2.5 h). The bacteria were transferred to 1.5 ml tubes and harvested by centrifugation at 1900 g for 5 min and then resuspended in PBS. The bacterial suspensions were adjusted to the required concentration for inoculation in BHI broth.

Bacterial doses of 1×10^5^ cfu/mouse were injected intraperitoneally (i.p.) with human holo-transferrin (Sigma, 10 mg/mouse) in a total volume of 500 µl to groups of 6–8-week-old wild-type C57BL/6J and corresponding SR-A^−/−^ knock-out inbred female mice. At the time of infection, the actual dose delivered to each group of mice was determined by serial dilution and replicate colony plating. At 18 h after the initial infection, mice were boosted i.p. with a further dose of human holo-transferrin (10 mg/mouse in 200 µl PBS). The health of the animals was monitored and scored at regular time points according to the symptoms presented as follows: Healthy = 5, ruffled fur = 4, sticky eyes = 3, ruffled fur and sticky eyes = 2, immobile = 1. As soon as immobile mice were detected they were humanely killed. Scores were then collated and averaged for each group at the various time points [29]; (A. Gorringe, personal communication). Survival curves were also plotted and statistical significance determined using the Log-rank (Mantel Cox) test. Blood samples from the tail vein (5 µl) were taken at 20 h post-infection and serial dilutions plated to determine bacteraemia. All dilutions were made with PBS. At termination, blood was collected by cardiac puncture and spleens removed. Half of each spleen was fixed in 2% paraformaldehyde in HEPES-buffered isotonic saline for immunohistochemical analysis. The remaining spleen was homogenized and serial dilutions of spleen and blood were plated to determine bacteraemia. The remaining blood was separated by centrifugation and the plasma collected and frozen at −80°C for later use. All procedures involving animals were conducted according to the requirements of the United Kingdom Home Office Animals (Scientific Procedures) Acts, 1986.

### Immunohistochemistry

Fixed tissues were transferred to a solution of 0.1 M sodium phosphate buffer containing 20% sucrose, placed in Tissue-Tek OCT compound (VWR International Ltd., Lutterworth, UK) and snap-frozen in isopentane cooled by dry ice. Frozen sections were cut on a Leica cryostat (5 µm thick), collected onto 1.5% gelatinized slides, air dried for 1 hour and stored at −20°C. Sections were washed in 0.1% Triton X-100 and endogenous peroxidase activity was quenched by incubation in PBS containing 0.01 M glucose, 0.001 M sodium azide and 40 U glucose oxidase for 15 min at 37°C. 5% Normal rabbit serum was used as blocking agent for non-specific binding and avidin/biotin blocking agents (Vector Laboratories Ltd., Peterborough, UK) were employed according to the manufacturer's instructions. Sections were incubated for 60 min in the respective primary antibodies or isotype-matched controls, washed and incubated for 30 min with the respective affinity purified biotinylated secondary antibodies. Finally, sections were washed and incubated with the avidin-biotin peroxidase complex (ABC elite, Vector Laboratories Ltd., Peterborough, UK) for 30 min and staining visualised by incubation with 0.5 mg/ml diaminobenzidine (Polysciences Inc., Northampton, UK) and hydrogen peroxide in 10 mM imidazole. Sections were counterstained with 0.1% methyl green (Vector Laboratories Ltd. Peterborough, UK) and mounted in DPX (VWR International Ltd., Lutterworth, UK).

### Cytokine Analysis

The concentration of interleukin-6 (IL-6) in the plasma of infected mice was determined using an OptEIA Mouse IL-6 ELISA set (BD Biosciences, San Diego) according to the manufacturer's instructions.

### Competitive Index Assay

Groups of five 6–8-week-old female wild-type C57BL/6J and corresponding SR-A^−/−^ animals were infected with 1×10^6^ *N. meningitidis* MC58 (wild-type)+1×10^6^ *N. meningitidis* MC58-278-1220 (mutant) i.p, along with 10 mg human holo-transferrin. The two bacterial strains were individually grown overnight as before. Serial dilutions of the inoculum were also plated onto both BHI medium and BHI medium supplemented with kanamycin (which selects for MC58-278-1220 mutant bacteria), in order to verify the dose and ratio of wild-type to mutant bacteria. A second dose of 10 mg human holo-transferrin was injected i.p. at 18 h. The health of the animals was monitored as before. At termination, blood was collected by cardiac puncture and spleens removed. Spleens were homogenized and serial dilutions of blood and spleen samples were plated on BHI medium and BHI medium supplemented with kanamycin. Enumeration of wild-type bacteria and mutant bacteria allowed for the determination of the CI ratio between wild-type and mutant bacteria using the following formula: CI = (wild-type output/mutant output)/(wild-type input/mutant input). The statistical significance of the results was determined using the paired student's t-test.

## Results

### Construction and Evaluation *N. meningitidis* Mutant Strains

To investigate the importance of NMB0278, NMB0667 and NMB1220 for recognition of *N. meningitidis* by SR-A, plasmids were constructed containing each gene interrupted by insertion of a kanamycin or erythromycin resistance cassette. The constructs pT7-278-kan and pT7-667-ery resulted from a single PCR product from NMB0278 and NMB0667, respectively, cloned into pT7Blue and then interrupted with an antibiotic resistance cassette. It is interesting to note that when a similar approach was utilised with NMB1220, the initial pT7-1220 construct proved to be highly unstable and consequently a two step approach of cloning the 5′ and 3′ regions of the gene together with some flanking DNA was undertaken, which proved to be successful. The plasmids pT7-278-kan, pT7-667-ery and pT7-12220-ery were transformed into *N. meningitidis* strain MC58.

The serogroup B genome sequence contains two homologues of NMB0278, namely NMB0294 and NMB0407. To ensure that only NMB0278 had been disrupted, specific oligonucleotide primers were designed to bind within the *kanR* cassette and in the neighbouring gene to identify transformants containing a single, disrupted copy of NMB0278. This transformant was designated MC58-278.

The gene NMB0667 shows low levels of homology to a degenerate DNA methylase found elsewhere in the serogroup B genome (NMB1223), so primers designed to bind within the *ermC* cassette and in the neighbouring gene were utilised to identify transformants containing a single, disrupted copy of NMB0667. A number of transformants were screened using different combinations of oligonucleotide primers. For each transformant containing an interrupted copy of NMB0667, an intact copy of the gene was present in tandem, therefore we concluded that NMB0667 is an essential gene, and consequently we were unable to obtain a mutant neisserial strain deficient in this protein.

Double mutants where both NMB1220 and NMB0278 had been interrupted were constructed by transforming MC58-1220 with chromosomal DNA from MC58-278. The disruption of NMB0278 and NMB1220 was confirmed by the use of specific oligonucleotide primers as for the single mutants. This transformant was designated MC58-278-1220.

### Characterisation of NMB Mutants

**Figure 1:**
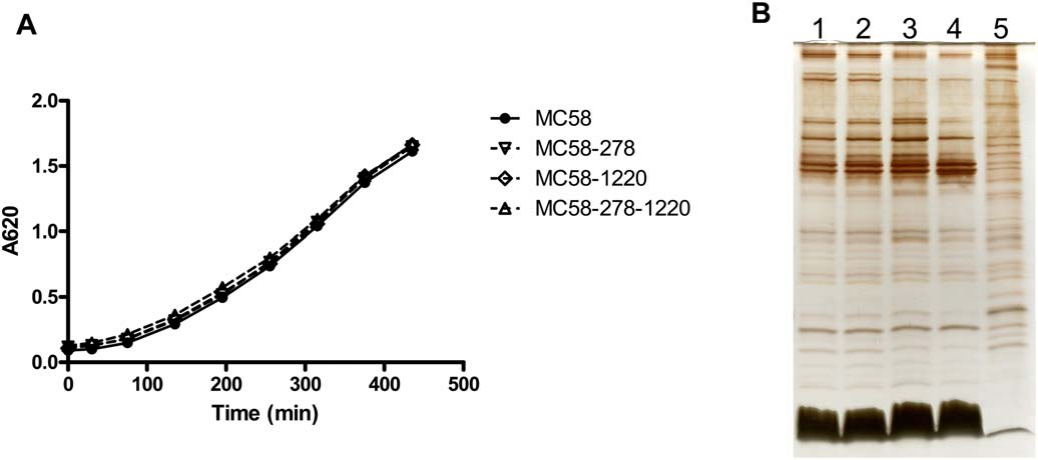
Growth characteristics of wild-type *N. meningitidis* MC58 bacteria and mutant bacteria. (A) *N. meningitidis* strains MC58, MC58-278, MC58-1220, and MC58-278-1220 bacteria were grown in Muller-Hinton broth supplemented with 0.25% (w/v) glucose. Growth was followed over an 8-h period and the OD_620_ was measured throughout. (B) Outer membranes were prepared from the parental strain and mutants following growth on BHI plates overnight, and separated by T-SDS-PAGE. Lane order: 1, strain MC58; 2, strain MC58-278; 3, strain MC58-1220; 4, strain MC58-278-1220; 5, whole cell lysate strain MC58.

The growth of the MC58-278, MC58-1220 and MC58-278-1220 *in vitro* was compared to that of the parental strain MC58. The bacteria were grown in Muller-Hinton broth in the same manner as for the preparation of inoculum for the mouse infection studies, except that growth was followed over an 8 h period and the OD_620_ was measured throughout (Figure 1A). All mutants exhibited growth curve patterns indistinguishable from the parent strain. Outer membranes were prepared from the mutants and parental strain following growth on BHI plates overnight and separated by T-SDS-PAGE (Figure 1B). The resulting profiles show that no significant differences were observed in the protein and LPS profiles. A comparison of the parental strain MC58 and the mutant strains showed no difference in the killing effect in pooled normal human serum (data not shown).

### Distribution of NMB0278, NMB0667, and NMB1220 across Different *N. meningitidis* Strains

In order to determine the distribution of these genes, a PCR screen was undertaken using primers specific not just for the gene under consideration, but also for its genomic location due to the homologous reading frames present for NMB0278 and NMB0667. 107 strains were screened using the following sets of primers; 278-out-f/278-out-r, 667-f/667-r, 667-f2/667-r and 1220-f/1220-r. This collection of strains was highly diverse and included representatives of disease and carriage isolates, along with well characterised reference strains. With the exception of one invasive disease strain, all gave a PCR product of the expected size for each of the three genes (refer to Table 3). The one anomalous strain demonstrated a PCR product for NMB0667 and NMB1220 but not for NMB0278. All three genes were determined to be present by this method in 7 *N. gonorrhoeae* isolates, however the distribution was found to vary considerably when other commensal species of *Neisseria* were analysed (data not shown).

**Table 3:** Screening of *N. meningitidis* strains for the presence of NMB0278, NMB0667, and MNB1220.

	NMB0278	NMB0667	NMB1220
Disease isolates from the UK	30/30	30/30	30/30
Immunotyping strains^1^	12/12	12/12	12/12
Sequenced strains^2^	3/3	3/3	3/3
Carriage collection from the Czech Republic [38]	17/17	17/17	17/17
Invasive disease samples from the UK [39]	25/26	26/26	26/26
Representatives from the MLST set [40]	11/11	18/18	18/18
Other laboratory strains	8/8	8/8	8/8

1 LPS immunotyping reference strains.

2 Genome sequenced serogroup A, B, and C strains.

A highly diverse collection of strains representative of disease and carriage isolates and including well-characterised reference strains were screened by PCR using specific oligonucleotide primers for the presence or absence of each of the three genes.

### Uptake of *N. meningitidis* Mutant Strains by Bone Marrow–Derived Macrophages

**Figure 2:**
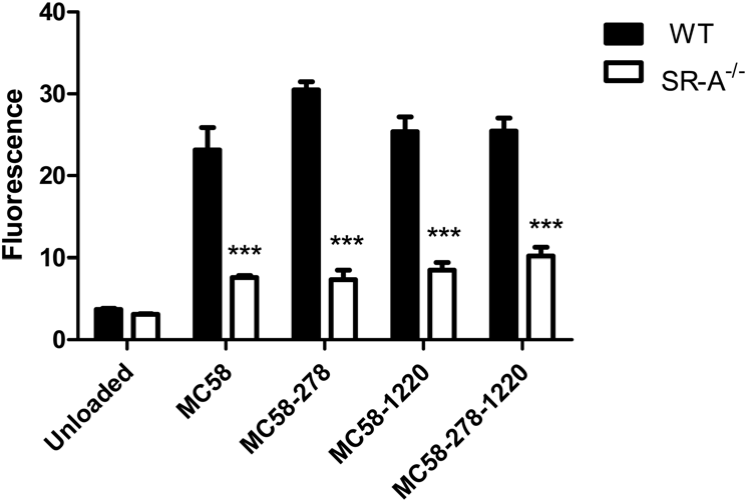
Uptake of wild-type *N. meningitidis* MC58 bacteria and mutant bacteria lacking NMB0278 (MC58-278), NMB1220 (MC58-1220), or both NMB0278 and NMB1220 (MC58-278-1220) by murine C57BL/6J wild-type (filled bars) and corresponding SR-A^−/−^ knock-out (open bars) bone marrow-derived macrophages. Macrophages were seeded on 6-well bacteriological plastic plates at 1×10^6^ cells/well and incubated with RdGnX-labelled ethanol-fixed MC58 or mutants lacking the surface proteins at an MOI of 20∶1 for 2 h at 37°C in the absence of serum. The Mϕ were washed to remove extracellular bacteria, detached using lidocaine/EDTA, and fixed with 4% formaldehyde before analysis by flow cytometry. Each measurement was performed in triplicate, and results are representative of at least three similar assays. The error bars represent standard deviation. Differences are statistically significant and *** indicates p<0.001.

To ascertain the *in vitro* macrophage uptake of wild-type bacteria compared to that of mutant bacteria, wild-type and SR-A^−/−^ bone marrow-derived macrophages were incubated at 37°C for 2 h with ethanol-fixed fluorescently labelled *N. meningitidis* MC58 or the mutant bacteria with deletions in either NMB0278, NMB1220 or both (MC58-278, MC58-1220 and MC58-278-1220), respectively, at an MOI of 20∶1 (Figure 2). Association of bacteria with macrophages was measured by flow cytometry. All the bacterial strains were taken up by wild-type macrophages, whereas uptake was reduced by at least 70% in macrophages lacking SR-A. However, no differences could be detected in the association of the mutant bacteria with wild-type macrophages. This could be attributed to the fact that there are multiple ligands for SR-A on *N. meningitidis* [18] and that the gene encoding at least one known ligand, NMB0667, could not be deleted. In addition, the strains still contained homologues for NMB0278.

### Meningococcal Septicaemia in Wild-Type versus SR-A^−/−^ Mice

**Figure 3:**
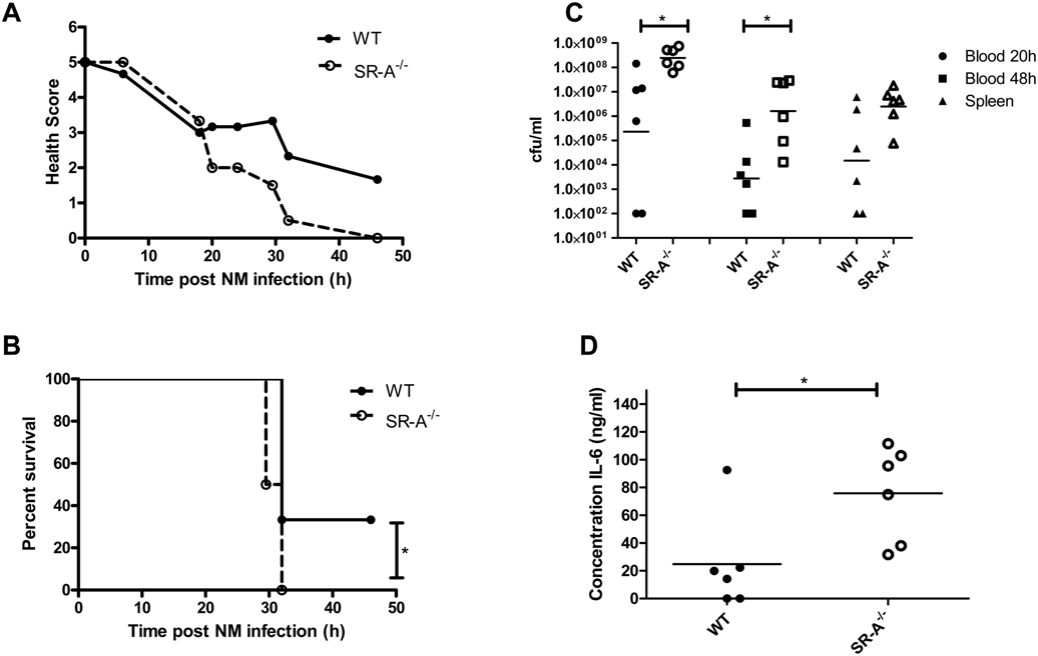
Six 6–8-week-old female C57BL/6J wild-type and corresponding SR-A^−/−^ mice were injected intraperitoneally with 1×10^5^ *N. meningitidis* MC58 and 10 mg human holo-transferrin per mouse. At 18-h post-injection a second dose of 10 mg human holo-transferrin was administered i.p. (A) The health of the animals was monitored regularly and scored at each time point according to the symptoms presented as follows: Healthy = 5, ruffled fur = 4, sticky eyes = 3, ruffled fur and sticky eyes = 2, immobile = 1. Scores were then collated for each group at each time point, averaged and plotted. (B) The survival of each group was plotted, and the statistical curve comparison was determined using the Log-rank (Mantel Cox) test (P = 0.0305). (C) Blood was collected from the tail vein at 20 h and by cardiac puncture at termination (48 h), and serial dilutions were plated on bacterial growth medium. Spleen segments were also collected, homogenized, and plated. Plates were incubated at 37°C, 5% CO_2_ overnight, and bacterial colonies counted. Counts for six individual animals in each group are plotted. Results were analysed using a two-tailed, unpaired t test, and the star indicates a statistically significant difference (P<0.05). (D) The concentration of interleukin-6 in the plasma from infected animals was measured at termination (48 h) using a standard ELISA assay. Individual measurements from 6 animals in each group are plotted. Results were analysed using a two-tailed, unpaired t test, and the star indicates a statistically significant difference (P<0.05).

Since no differences could be detected between wild-type and mutant bacteria in their interaction with SR-A *in vitro*, we set up an *in vivo* murine septicaemia model to investigate the role of SR-A in the clearance of *N. meningitidis*. First, we tested several bacterial doses in wild-type and SR-A^−/−^ animals to determine the intraperitoneal (i.p.) dose required to establish bacteraemia in the blood and spleen, without causing rapid death of the animals, so that they could be monitored over time. From these experiments we selected a dose of 1×10^5^ cfu/mouse (data not shown). Groups of six age-matched female C57BL/6J wild-type and corresponding SR-A^−/−^ knock-out mice were injected i.p. with 1×10^5^ *N. meningitidis* MC58 cfu/mouse and 10 mg human holo-transferrin as a bacterial iron source. *N. meningitidis* requires iron for growth and is unable to sequester iron from murine transferrin [30],[31]. A second dose of human holo-transferrin was administered i.p. at 18 h to maintain available iron levels in the blood. Animals were monitored regularly over a period of 48 h for symptoms of septicaemia. Each individual was assigned a health score at each time point, according to the severity of symptoms (animals with a score of 5 were healthy, and a score of 1 was assigned when they were immobile, refer to materials and methods). Scores were then collated and averaged for the group, and the results plotted to provide a health curve (Figure 3A).

Although animals in both groups exhibited symptoms of septicaemia, wild-type animals remained healthier, particularly after the second injection of iron, which would support bacterial proliferation. Survival curves (Figure 3B) also show that all the SR-A^−/−^ animals had died at 32 h, while 33% of the wild-type animals survived beyond 48 h and recovered to full health. Overall, SR-A^−/−^ animals showed more rapid deterioration of health and died more quickly than did wild-type animals. This could be correlated with the observation that SR-A^−/−^ animals had higher levels of bacteraemia in their blood at both 20 h and 48 h than did wild-type animals (Figure 3C). Although there seemed to be a trend towards higher bacterial levels in the spleens of SR-A^−/−^ animals, the differences in bacteraemia between the two strains were not statistically significant. We also measured levels of interleukin-6 (IL-6) in plasma from blood collected at 48 h. SR-A^−/−^ animals consistently showed significantly higher levels of the pro-inflammatory cytokine IL-6 than wild-type animals.

**Figure 4:**
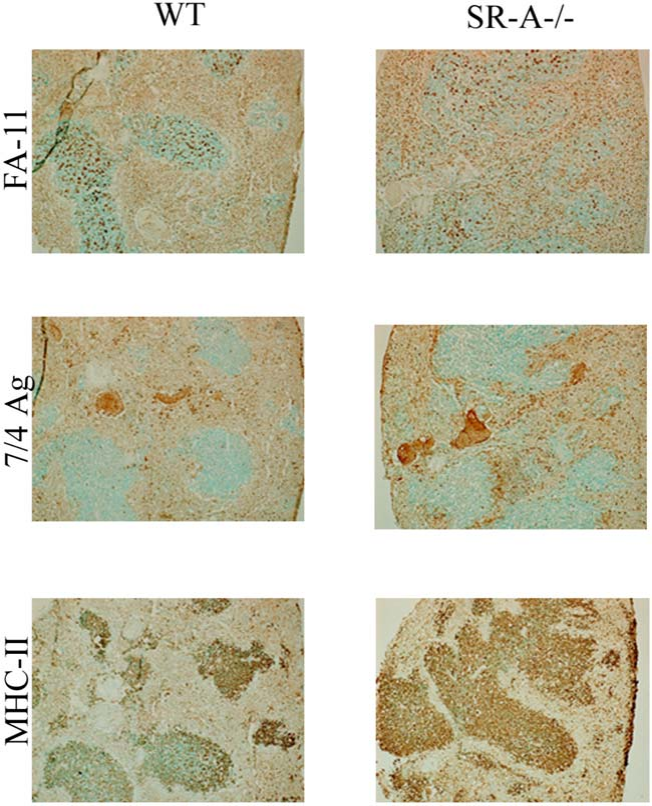
Intact spleen segments from infected animals were fixed in paraformaldehyde, embedded in OCT, sectioned, and stained with various antibodies for the infiltration of macrophages (FA-11), polymorphonuclear neutrophils, and activated monocytes (7/4Ag) and activated macrophages (MHC-II), as described in the text. All data are representative of at least three individual experiments.

Spleen sections were also analysed by immunohistochemistry with three antibodies, FA-11 (an intracellular macrophage marker, staining macrosialin [CD68]), 7/4 Ag (a neutrophil and monocyte marker) and MHC-II (an antibody recognising the major histocompatibility class II molecule, a marker expressed on resident dendritic cells and activated macrophages). FA-11 (macrosialin) is a pan-macrophage marker, indicating the macrophage infiltration into the spleen. Staining with MHC-II shows that the macrophages are activated. The 7/4 Ag staining shows the infiltration of activated monocytes and polymorphonuclear neutrophils (Figure 4). Therefore all the splenic samples showed a high number of infiltrating activated macrophages and neutrophils.

### Effect of *N. meningitidis* Mutants *In Vivo*

**Figure 5:**
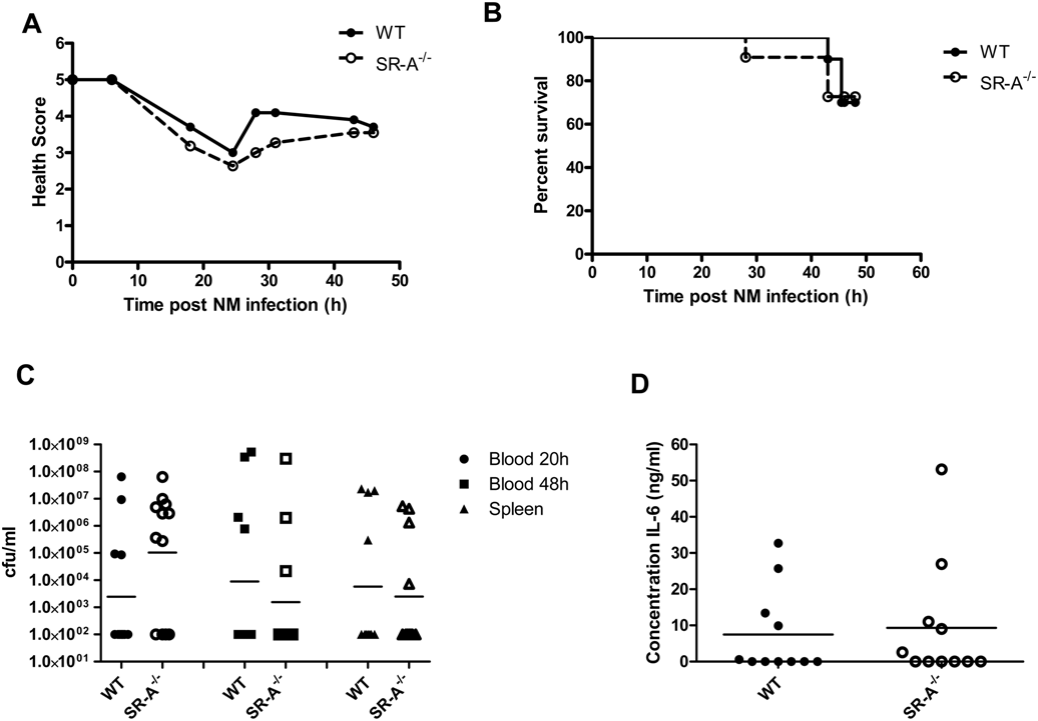
Eleven 6–8-week-old female C57BL/6J wild-type and corresponding SR-A^−/−^ mice were infected intraperitoneally with 1×10^5^ *N. meningitidis* MC58-278-1220 and 10 mg human holo-transferrin per mouse. At 18 h post-injection a second dose of 10 mg human holo-transferrin was administered i.p. (A) The health of the animals was monitored regularly and scored at each time point according to the symptoms presented as before. Scores were then collated for each group at each time point, averaged, and plotted. (B) The survival of each group was plotted and the statistical curve comparison was determined using the Log-rank (Mantel Cox) test. (C) Blood was collected by tail vein bleed at 20 h, and at termination (48 h) blood was collected by cardiac puncture and spleens were collected and homogenised. Serial dilutions of blood and spleen were plated on bacterial growth medium, incubated, and bacterial colonies were counted. Counts for 11 individual animals in each group are plotted. Results were analysed using a two-tailed, unpaired t test (P>0.05). (D) The concentration of interleukin-6 in the plasma was measured at termination (48 h) using a standard ELISA assay. Individual measurements from 11 animals in each group are plotted. Results were analysed using a two-tailed, unpaired t test (P>0.05). All data are representative of at least three individual experiments.

Next, we tested the mutant bacteria lacking both SR-A ligands, NMB0278 and NMB1220, for their ability to establish septicaemia in wild-type and SR-A^−/−^ animals. Groups of eleven age-matched female C57BL/6J wild-type and corresponding SR-A^−/−^ knock-out mice were injected i.p. with 1×10^5^ *N. meningitidis* MC58-278-1220 cfu/mouse and 10 mg human holo-transferrin as before. Animals were monitored regularly, assigned health scores and their survival plotted (Figure 5A and 5B). Animals in both groups exhibited symptoms of septicaemia, however differences observed between wild-type and SR-A^−/−^ animals when infected with wild-type bacteria, were absent. Overall both mouse strains showed fewer symptoms of septicaemia and had a higher survival rate when infected with mutant MC58-278-1220 bacteria (Figure 5A and 5B). No statistically significant differences were observed in the levels of bacteraemia in either the blood or the spleen between wild-type and SR-A^−/−^ animals (Figure 5C). IL-6 levels were also not statistically significantly different between wild-type and SR-A^−/−^ animals and were lower overall than in animals infected with wild-type bacteria (Figure 5D). Therefore the SR-A-mediated effects observed when mice were infected with wild-type bacteria were abrogated for mutant bacteria lacking two SR-A ligands.

### *In Vivo* Competition Assay

When MC58-278-1220 mutant bacteria were injected into wild-type and SR-A^−/−^ mice, we observed not only the abrogation of the SR-A-mediated effect, but also that mutant bacteria seemed to cause less severe symptoms of septicaemia and animals had a higher survival rate. To test whether this was a bona fide observation, we employed a competition assay to determine whether wild-type bacteria would out-compete mutant bacteria *in vivo*. To obtain a bacterial count for wild-type and mutant bacteria in each case, we injected 1×10^6^ wild-type MC58 and mutant MC58-278-1220 bacteria i.p. at a ratio of 1∶1. The respective bacterial numbers in blood and spleen were determined by replicate plating of serial dilutions on BHI medium and BHI medium supplemented with kanamycin, which would select for mutant bacteria carrying the kanamycin resistance cassette used to disrupt the gene encoding NMB0278. Table 4 shows the competitive indices in wild-type and SR-A^−/−^ animals. A competitive index of 6.891 and 5.090 was obtained for bacterial counts from blood and spleen from wild-type animals, respectively. This indicates that more wild-type bacteria remained than did mutant bacteria. In SR-A^−/−^ animals, a competitive index of 2.781 and 1.660 was obtained from blood and spleen, respectively.

**Table 4:** Competitive Index of wild-type *N. meningitidis* MC58 versus mutant *N. meningitidis*-278-1220.

Mouse Strain	Bacteria Isolated from:	Competitive Index^1^	P Value^2^
C57BL/6J	Blood	6.891	0.0414*
	Spleen	5.090	0.0343*
C57BL/6J-SR-A^−/−^	Blood	2.781	0.0507
	Spleen	1.660	0.0231*

1 (wild-type output/mutant output)/(wild-type input/mutant input).

2 Paired t test.

*: Values marked with an asterisk are statistically significant with a 95% confidence interval.

## Discussion

In this study we evaluated the *in vivo* role of SR-A in meningococcal septicaemia induced by *N. meningitidis* MC58 (serogroup B), and ascertained the role of the *N. meningitidis* surface proteins, previously identified to be ligands for SR-A [18]. The neisserial surface protein ligands were NMB0278, NMB0667 and NMB1220. NMB1220 has been shown to be surface expressed and similar experiments showed the surface expression of NMB0278 and NMB0667 [32] and personal communication). The function of these proteins is unknown, however they show some homology to proteins identified in other bacterial species. Sequence analysis suggests that NMB0278 has homology to *E. coli* DsbA, which functions in disulphide bond formation [33]. Interestingly, the DsbA protein of *Haemophilus influenzae*, another obligate human pathogen colonising the nasopharynx, has recently been shown to be a virulence factor [34]. The C-terminus of NMB0667 has 20% homology with the ZipA protein from *E. coli*, which is involved in septum formation during cell division [35]. NMB1220 belongs to the stomatin/Mec-2 protein family, which are oligomeric lipid raft-associated integral proteins that regulate the function of ion channels and transporters. We constructed deletion mutants in *N. meningitidis* MC58 for all three proteins, respectively, and also generated a double mutant lacking NMB0278 and NMB1220. We employed several approaches to delete NMB0667, however this mutation proved lethal, which may be linked to the possible role of NMB0667 in septum formation during cell division. All the mutant strains showed no deficiency in growth characteristics or LPS and protein profile. Through PCR analysis, we showed that the genes encoding these proteins are present in a wide variety of neisserial strains, including invasive disease and carriage strains. Closely related homologues are also present in other bacteria, making them ideal PAMPs (pathogen-associated molecular patterns) and targets for PRRs. It should however be noted that since the genes encoding these proteins are also present in non-pathogenic bacteria and commensal strains, the term “PAMPs”, though commonly used in this context, could be considered a misnomer.

*In vitro* uptake of the mutant bacteria by bone marrow-derived macrophages from wild-type and SR-A^−/−^ animals did not show any differences when compared to wild-type bacteria. This could be attributed to the fact that at least one other SR-A ligand, NMB0667, was present, along with two NMB0278 homologues, which could mediate recognition and uptake. Therefore, we studied the *in vivo* role of SR-A in a murine model for meningococcal septicaemia. We injected *N. meningitidis* MC58 into wild-type C57BL/6J and corresponding SR-A^−*/*−^ mice and monitored the manifestation of symptoms of septicaemia (ruffled fur, sticky eyes), as well as the survival of the animals. The health of SR-A^−/−^ animals consistently deteriorated more rapidly and there was a 33% difference in survival when compared to wild-type animals. Analysis of blood samples taken at 20 h post-infection and at termination showed that SR-A^−/−^ animals had higher levels of bacteraemia and the pro-inflammatory cytokine, IL-6. Induction of IL-6 is commonly associated with meningococcal septicaemia in humans [36]. Although not statistically significant, spleen samples also indicated higher bacteraemia for SR-A^−/−^ animals, and splenic sections revealed infiltration of activated macrophages, monocytes and neutrophils. Therefore, in this model, SR-A played an important role in clearance of *N. meningitidis* MC58. This is the first report of the *in vivo* importance of SR-A in a model using a Gram-negative pathogenic organism.

Subsequently, we injected the mutant bacteria lacking both NMB0278 and NMB1220 at the same dose i.p. into wild-type and SR-A^−/−^ mice and monitored them as before. Interestingly, the differences observed between the mouse strains when infecting with wild-type bacteria were abrogated for mutant bacteria lacking the two SR-A ligands, and overall less severe symptoms were observed. Furthermore, the levels of bacteraemia and IL-6 between wild-type and SR-A^−/−^ animals did not differ significantly. Therefore the surface proteins NMB0278 and NMB1220 are at least partially implicated in the SR-A-mediated effects observed for wild-type bacteria. We previously showed that NMB0278 and NMB1220 are also Toll-like receptor (TLR) agonists, and that SR-A was required for full activation of TLR pathways (Plüddemann et al. JII, in press). Although SR-A ligation does not directly mediate cytokine induction, the overall lower cytokine levels in animals infected with mutant bacteria could be linked to the absence of NMB0278 and NMB1220. The competitive index assay confirmed that mutant bacteria were cleared more readily, proving that this observation was not due to inter-experimental variations. These data indicate a role for SR-A in clearance of *N. meningitidis* and development of symptoms of septicaemia. Thus far, the development and progression of septicaemia has mainly been linked to LPS, however these data indicate an additional role of neisserial surface protein recognition in this process.

It is clear that the i.p. mouse challenge model described here does not represent the natural pathogenesis of neisserial disease, however it does model the overwhelming septicaemia that is characteristic of invasive meningococcal disease. Since SR-A is not expressed on polymorphonuclear neutrophils, our results signify an important role for macrophages and SR-A in the development and progression of meningococcal septicaemia.
